# Performance of mutiple blood‐based biomarkers used in community Alzheimer’s disease screening via a new ultrasensitive micro‐array immunoassay platform

**DOI:** 10.1002/alz.091162

**Published:** 2025-01-09

**Authors:** Peng Cheng, Minchao Zhang, Hao Chen, Hui Li

**Affiliations:** ^1^ ColorTech (Suzhou) Biotechnology Co., Ltd., suzhou, JIANGSU China

## Abstract

**Background:**

AD is a neurodegenerative disease characterized by progressive cognitive dysfunction and behavioral impairment. The use of biomarkers for early detection of AD is crucial for developing potential treatments. The benefit of using blood‐based biomarkers in screening for AD is that the collection of blood is less invasive than the collection of cerebrospinal fluid, and less costly than neuroimaging. Numerous studies have reported that certain biomarkers are good candidates for AD diagnostics. However, most of the studies utilized clinical cohorts which are less heterogenous than the community‐based population. In China, the number of AD patients is growing rapidly, which poses a considerable burden on society and families. There is a need to establish predictive values of blood‐based biomarkers. This study evaluates the potential of an ultrasensitive micro‐array immunoassay to measure plasma biomarkers and further used in screening AD among the community‐based population in China.

**Method:**

An ultrasensitive micro‐array immunoassay, with a limit of detection (LOD) under 100 fg/mL was developed to measure AD related biomarkers. Blood‐based biomarkers (pTau181, Aβ1‐42, Aβ1‐40 etc.) were measured using this method. Above 1500 plasma samples (age>55) were analyzed. Some of the samples had Aβ‐PET data. Results were adjusted for age, sex and year of education.

**Result:**

The population that involved in this study are retired hospital employees. The levels of the blood‐based biomarkers were measured. The level of these biomarkers showed associations with age, sex, education level, BMI, as well as other medical conditions (hypertension, diabetes, etc.) The associations of these variables were used to construct adjusted models. Comparison of different models were performed to evaluate the predictive performance. Model PAV II was chosen as the best model for predicting AD.

**Conclusion:**

By using an ultrasensitive micro‐array immunoassay, the levels of blood‐based biomarkers were evaluated. Models for predicting AD were constructed and evaluated. Model PAV II involve the levels of biomarkers, medical conditions and other variables showed the best result for predicting AD. The results from the community‐based population indicate that biomarkers in blood have high potential to be used as a screening tool for the detection of AD.

